# Dietary Fiber Intake May Influence the Impact of *FTO* Genetic Variants on Obesity Parameters and Lipid Profile—A Cohort Study of a Caucasian Population of Polish Origin

**DOI:** 10.3390/antiox10111793

**Published:** 2021-11-09

**Authors:** Przemyslaw Czajkowski, Edyta Adamska-Patruno, Witold Bauer, Urszula Krasowska, Joanna Fiedorczuk, Monika Moroz, Maria Gorska, Adam Kretowski

**Affiliations:** 1 Clinical Research Centre, Department of Nutriomics, Medical University of Bialystok, Jana Kilinskiego 1, 15-089 Bialystok, Poland; witold.bauer@umb.edu.pl (W.B.); urszula.krasowska@umb.edu.pl (U.K.); adamkretowski@wp.pl (A.K.); 2 Clinical Research Centre, Medical University of Bialystok Clinical Hospital, Marii Sklodowskiej-Curie 24A, 15-276 Bialystok, Poland; j.fiedorczuk@wp.pl (J.F.); monika_bakun@wp.pl (M.M.); 3 Department of Endocrinology, Diabetology and Internal Medicine, Medical University of Bialystok, Jana Kilinskiego 1, 15-089 Bialystok, Poland; mgorska25@wp.pl

**Keywords:** *FTO* gene, dietary fiber, gene–diet interaction, glucose metabolism, macronutrients, obesity

## Abstract

Genetic and environmental factors play a key role in the development of obesity. The aim of this study was to explore the potential effect of fat mass and obesity-associated (*FTO*) rs3751812, rs8050136, rs9939609, rs6499640, rs8044769, and rs7190492 genotypes and dietary fiber intake on the obesity-related parameters and lipid profile in the Polish population. We selected 819 Polish Caucasian adult subjects (52.5% female and 47.5% male) for a final gene–diet interaction analysis, with mean BMI 28.5 (±6.6) kg/m^2^. We performed measurements of anthropometric parameters, total body fat content and distribution, and blood glucose, insulin, and lipid concentrations. Daily fiber intake was analyzed based on 3-day food-intake diaries, and daily physical activity was evaluated based on the International Physical Activity Questionnaire—Long Form. Our study shows that carriers of the GG genotype (rs3751812), CC genotype (rs8050136), and GG genotype (rs6499640) presented lower hip circumference if daily fiber intake was above 18 g per day. Additionally, GG genotype (rs3751812) and CC genotype (rs8050136) carriers showed surprisingly higher total cholesterol and LDL-cholesterol levels when they were stratified to the group with higher than median fiber intake. The results of this study highlight that high-fiber diets may positively affect anthropometric parameters but may also worsen lipid profile dependent on the *FTO* genotype.

## 1. Introduction

The prevalence and development of obesity has increased substantially worldwide [1]. It has been established that obesity is a leading factor for diabetes [2] and cardiovascular disease prevalence [3]. In addition, abnormalities in serum lipids and their metabolism are one of the major risk factors of cardiovascular diseases [4]. One of the major strategies that has been attempted to address this is a balanced diet that fulfils all nutritional needs, connected with genome-customized recommendations.

The fat mass and obesity-associated (*FTO*) gene has been reported as the gene with the strongest significant correlation with obesity [5]. The associations between *FTO* genetic variants, dietary factors, and metabolic consequences are still under investigation. However, it has been postulated that associations of some *FTO* variants with obesity can occur due to their influence on dietary intake [6]. Moreover, in our previously published results, we observed that dietary carbohydrate, protein, and fat intake may modulate the impact of *FTO* genetic SNPs on obesity and obesity-related metabolic consequences [7]; therefore, we decided to perform additional analyses, including on dietary fiber intake.

Dietary fiber is a non-digestible carbohydrate, derived mainly from fruits and vegetables. Moreover, dietary fiber antioxidant capacity is mainly based on the effect of bioaccessibility and bioavailability of natural antioxidants, including polyphenol compounds, in the diet [8]. Unfortunately, this interaction of dietary fiber and other components of diet could result in retarded absorption of micro- and macronutrients in the small intestine. Nevertheless, it has been shown that dietary fiber intake plays an important role in the improvement of parameters such as glycemic response [9] and plasma lipid profile [10].

The purpose of this study is to evaluate whether daily fiber intake can modify the association between genetic variations of the *FTO* gene and obesity in the Polish Caucasian population. To the best of our knowledge, this study is the first to examine associations between dietary fiber intake and *FTO* genetic variants in the Polish population, based on gene–diet interactions.

## 2. Materials and Methods

### 2.1. Participants of the Study Cohort

The study group comprised 819 participants recruited from 1549 Polish-origin Caucasian volunteers (18–79 years old) of the 1000PLUS Cohort Study (registered at www.clinicaltrials.gov as NCT03792685), described previously [7,11,12,13]. Participants who previously took diet supplements, medicines (weight loss, anti-diabetic, lipid-lowering, etc.), or other chemical and natural-based substances that could affect the results were eliminated from this study. Individuals who reported metabolic, endocrine, hepatic, or gastrointestinal disorders or who had bariatric surgery, which could have an impact on the study, were excluded from the study analysis as well. Subjects who previously took treatment, or who followed any special diet or dietary pattern (vegan, vegetarian, etc.), and others who met the exclusion criteria mentioned above, were not included in the analysis.

### 2.2. Anthropometric Measurements and Body Composition Analysis

In all subjects, we recorded the body weight and height using a standardized method [14]. Using the bioelectrical impedance method (InBody 220, Biospace, Seoul, Korea), we analyzed total body composition (fat mass, fat-free mass, and skeletal muscle mass). Body fat distribution analysis, including determination of the percentage of total body fat, visceral adipose tissue (VAT), subcutaneous adipose tissue (SAT), and VAT/SAT ratio, was performed by the multi-frequency bioimpedance method (MaltronBioScan 920-2, Maltron International Ltd., Rayleigh, UK).

### 2.3. Oral Glucose Tolerance Test (OGTT) Performance

The OGTTs were performed in non-diabetic participants according to the World Health Organization (WHO) recommendations, using a 75 g oral glucose dose. The subjects were instructed to fast for 8–12 h prior to the tests, but not to restrict carbohydrate intake 3 days before the test. Blood was collected at 0, 30, 60, and 120 min after glucose load. Glycemia and insulin levels were measured in all study participants without a history of diabetes.

### 2.4. Blood Collection and Biochemical Analysis

Blood samples were obtained and collected to evaluate the concentrations of plasma glucose, insulin, total cholesterol, low-density lipoprotein (LDL), high-density lipoprotein (HDL), triglyceride (TG), and hemoglobin A1c (HbA1c). The samples were stored in accordance with the kit instructions until testing at −20 or −80 °C. The samples were prepared for testing in accordance with the instructions provided with the laboratory kit. Concentrations of plasma glucose were measured by the hexokinase enzymatic colorimetric assay (Cobas c111, Roche Diagnostics Ltd., Risch-Rotkreuz, Switzerland). Serum insulin concentrations were evaluated by immunoradiometric assay (INS-Irma, DIASource S.A., Ottignies-Louvain-la-Neuve, Belgium; Wallac Wizard 1470 Automatic Gamma Counter, PerkinElmer Life Sciences, Turku, Finland). Concentrations of lipids were evaluated by enzymatic colorimetric assay using commercially available kits (Cobas c111, Roche Diagnostic Ltd., Risch-Rotkreuz, Switzerland). HbA1c levels were assessed using HPLC (high performance liquid chromatography; D-10 Hemoglobin Testing System, Bio-Rad Laboratories Inc., Hercules, CA, USA by France, Bio-Rad, Marnes-la-Coquette).

### 2.5. Calculations

Body mass index (BMI) was calculated using the following formula: body weight (kg) divided by height (m) squared. The waist–hip ratio (WHR) was determined by dividing waist circumference by hip circumference. The VAT/SAT ratio was calculated by dividing visceral adipose tissue content by subcutaneous adipose tissue content. In order to evaluate insulin resistance, we performed the homeostasis model assessment (HOMA-IR), following the standard formula: (fasting plasma glucose concentration (mmol/L)) × (fasting insulin concentration (µU/mL))/22.5. Homeostatic model assessment of β-cell function (HOMA-B) was determined using the following formula: 20 × fasting insulin (μIU/mL)/fasting glucose 100 (mmol/mL) − 3.5. The metabolic equivalent (MET, min per week) was calculated using the following formula: (MET level) × (minutes of activity) × (events per week).

### 2.6. Daily Physical Activity and Dietary Intake Analyses

Daily physical activity was estimated using the International Physical Activity Questionnaire—Long Form (IPAQ-LF), which is a self-administered questionnaire [15]. The results of the questionnaire were used to assess the level of physical activity, expressed as MET values. Each subject was stratified as having a low, moderate, or high level of physical activity.

We conducted a 3-day food-intake diary analysis. Participants were instructed to weigh food and estimate portion sizes based on provided color photograph albums of portion sizes. Daily carbohydrate, protein, fat, and fiber intake were estimated using Dieta 6.0 software (National Food and Nutrition Institute, Warsaw, Poland). Dieta 6.0 software is used to calculate the nutritional value of food and diets based on the tables of the nutritional value of local food products. In order to study the interactions between genetic factors and diet, study participants were divided into 2 quantiles based on average daily fiber intake: lower and higher than median dietary fiber intake (≤18 g a day and >18 g a day of total fiber intake, respectively).

### 2.7. Genetic Analysis

We selected and genotyped 6 previously identified *FTO* SNPs in rs3751812 (G > T), rs8050136 (A > C), rs9939609 (T > A), rs6499640 (G > A), rs8044769 (C > T), and rs7190492 (A > G), based on the validated catalog of published genome-wide association studies [16]. DNA was extracted from peripheral blood leukocytes using a classical salting-out method. The SNPs were genotyped with TaqMan SNP technology from a ready-to-use human assay library (Applied Biosystems, Beverly, MA, USA) using a high-throughput genotyping system, OpenArray (Life Technologies, South San Francisco, CA, USA). SNP analysis was performed in duplicate, following the manufacturer’s instructions. As a negative control, we used a sample without a template. The negative control was applicable in measuring any false positive signal caused by contamination.

### 2.8. Ethics Statement

The study methods were carried out in accordance with the ethical standards of the responsible committee on human experimentation (Ethics Committee of the Medical University of Bialystok, Poland, R-I-002/35/2009) and with the Helsinki Declaration of 1975. Written informed consent was obtained from all participants included in the study.

### 2.9. Statistical Analysis

Numerical data were summarized with number of observations (N), arithmetic mean, and standard deviation (SD). For categorical data, the number of observations and frequency (percentage) are presented. Study participants were divided into quantiles based on average daily fiber intake, with the thresholds set as the median value of each parameter. Risk genotypes of the 6 previously identified *FTO* SNPs were predefined based on the literature and our previous findings. Comparisons of the allelic and genotypic frequencies as well as odds ratio calculations in this study were not included because of the relatively small sample size. Continuous parameters were tested for normality with Shapiro–Wilk’s test and by visual inspection. Homogeneity of variance across groups was studied using Levene’s test. Nonparametric tests were used for response variables that failed the mentioned statistical tests. Differences between selected parameters and dietary groups were then compared using analysis of variance (ANOVA) or Kruskal–Wallis test for numerical variables, with either Tukey’s or Dunn’s post hoc test with Holm p-value adjustment (in case multiple pairwise tests were performed, or when there were multiple grouping variables, as presented in tables and figures), and chi-squared test for categorical variables. The statistical significance level was set at <0.05 for all 2-sided tests and multivariate comparisons. All calculations were prepared in R (version 4.0.3).

## 3. Results

**Figure 1:**
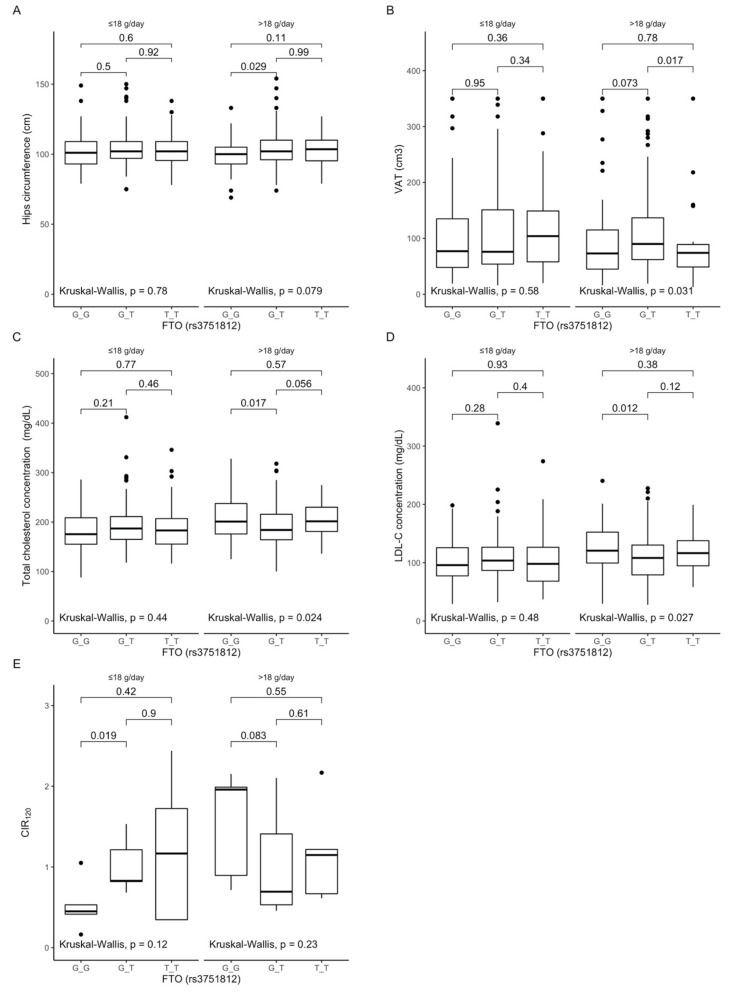
Association of the *FTO* rs3751812 genotypes with (**A**) Hip circumference (cm); (**B**) Visceral adipose tissue (VAT) (cm^3^); (**C**) Total cholesterol concentration (mg/dL); (**D**) LDL-C concentration (mg/dL); (**E**) CIR 120 by dietary fiber intake strata: ≤18 g/day and >18 g/day.

**Figure 2:**
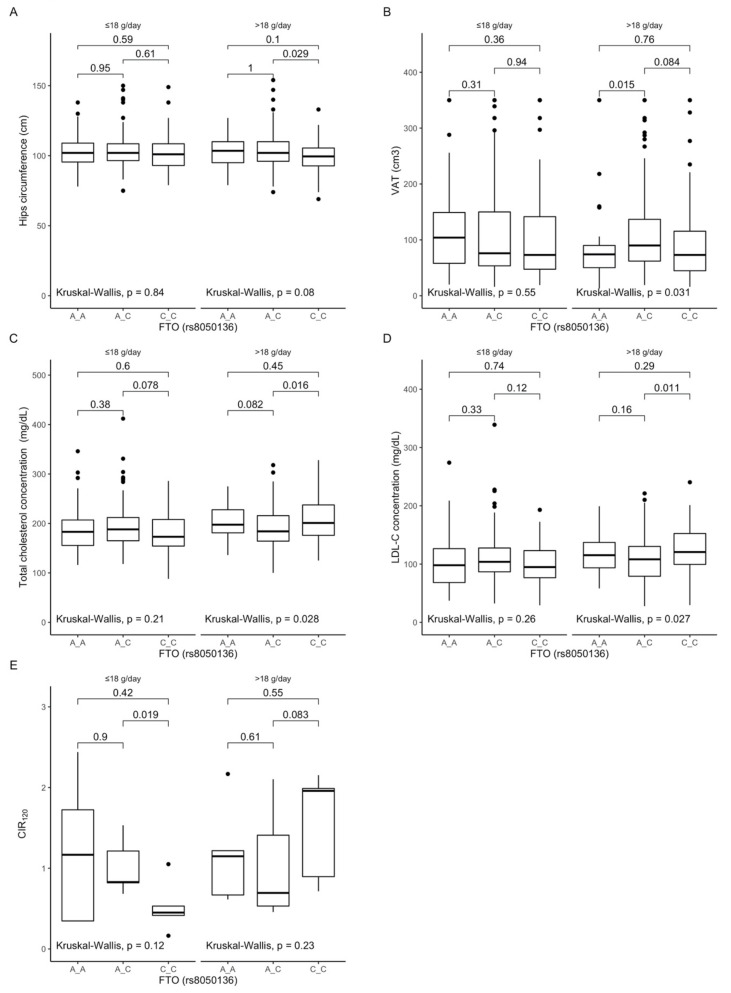
Association of the *FTO* rs8050136 genotypes with (**A**) Hip circumference (cm); (**B**) Visceral adipose tissue (VAT) (cm^3^); (**C**) Total cholesterol concentration (mg/dL); (**D**) LDL-C concentration (mg/dL); (**E**) CIR 120 by dietary fiber intake strata: ≤18 g/day and >18 g/day.

**Figure 3:**
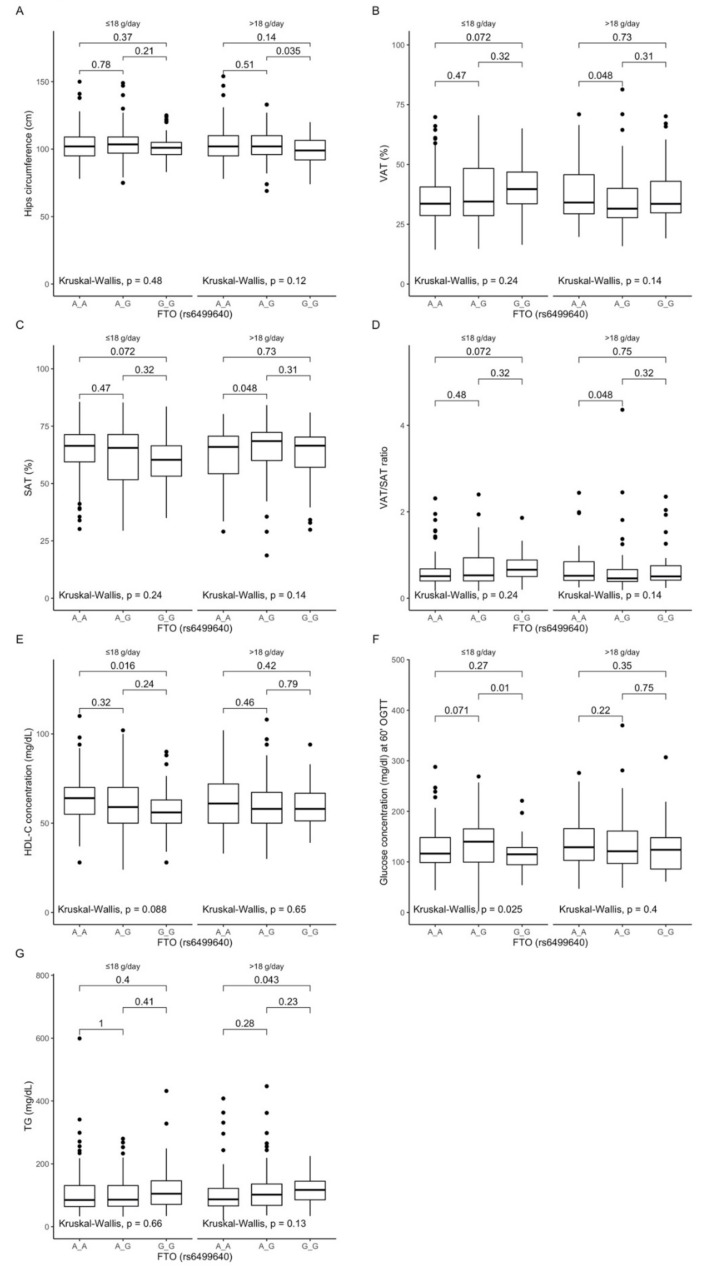
Association of the *FTO* rs6499640 genotypes with (**A**) Hip circumference (cm); (**B**) Visceral adipose tissue (VAT) (cm^3^); (**C**) Subcutaneous adipose tissue (SAT) (%); (**D**) VAT/SAT ratio; (**E**) HDL-C concentration (mg/dL); (**F**) Glucose concentration (mg/dL) at 60 min of OGTT; (**G**) Triglycerides (TG) (mg/dL) by dietary fiber intake strata: ≤18 g/day and >18 g/day.

**Figure 4:**
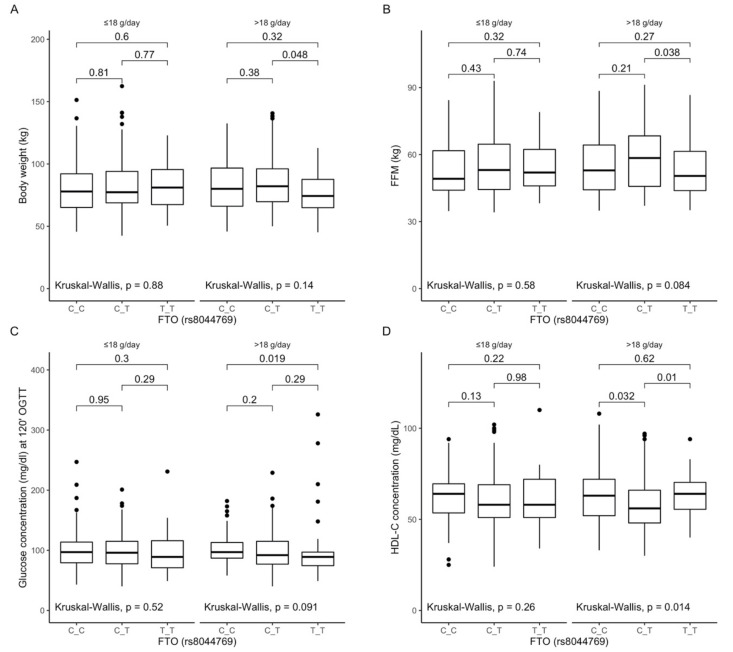
Association of the *FTO* rs8044769 genotypes with (**A**) Body weight (kg); (**B**) Fat-free mass content (FFM) (kg); (**C**) Glucose concentration (mg/dL) at 120 min of OGTT; (**D**) HDL-C concentration (mg/dL) by dietary fiber intake strata: ≤18 g/day and >18 g/day.

**Figure 5:**
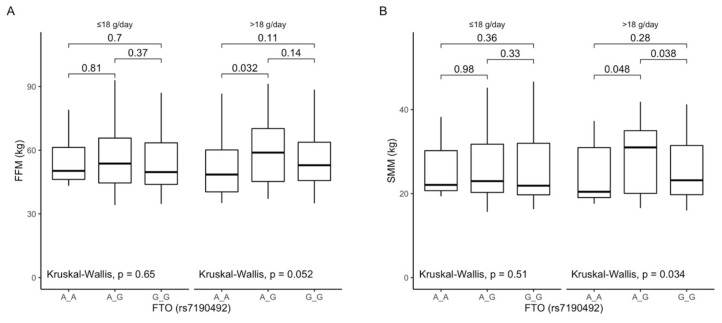
Association of the *FTO* rs7190492 genotypes with (**A**) Fat-free mass content (FFM) (kg); (**B**) Skeletal muscle mass content (SMM) (kg) by dietary fiber intake strata: ≤18 g/day and >18 g/day.

Data from 819 participants (52.5% female and 47.5% male) were included in the gene–diet interaction analysis, as described previously [7]. The mean age of the subjects was 42.1 (±14.5) years, and the mean BMI was 28.5 (±6.6) kg/m^2^. Among the individuals, 33.9% had a BMI of <25.00 kg/m^2^, 34.5% were overweight with a BMI of ≥25.00 and <30.00 kg/m^2^, and 31.6% were obese with a BMI of ≥30.00 kg/m^2^. Among the study population, 411 individuals (50.2%) were identified as having prediabetes or diabetes. Of these subjects, 109 participants (13.3%) had a previous history of prediabetes or diabetes, 56 participants (6.8%) previously took anti-diabetic drugs and 47 participants (5.7%) were being treated with lipid-lowering medications. The clinical characteristics, stratified by investigated genotypes, are presented in Table 1, Table 2 and Table 3. No significant deviation from the Hardy–Weinberg equilibrium was reported for any of the investigated SNPs (*p* > 0.05). Among the investigated FTO SNPs, some of the loci were in strong link disequilibrium (D’ = 1.0 for rs8050136 and rs9939609) [17]; thus, we present the results for one of them: rs8050136. We analyzed the same parameters for all of the investigated SNPs, however, in Figure 1, Figure 2, Figure 3, Figure 4 and Figure 5 we present only statistically significant results.

**Table 1:** Characteristics of participants stratified by rs3751812 and rs8050136 genotypes.

	rs3751812				rs8050136			
	G/G	G/T	T/T	*p* Value	C/C	A/C	A/A	*p* Value
N	211	420	181		209	424	182	
Genotype frequency	0.26	0.52	0.22	>0.05	0.26	0.52	0.22	>0.05
Age (years)	40.5 (14.2)	41.2 (14.7)	39.5 (14.3)	0.33	40.2 (14.1)	41.3 (14.8)	39.4 (14.3)	0.24
BMI (kg/m^2^)	27.6 (6.0)	28.7 (6.8)	28.9 (6.8)	0.060	27.6 (6.1)	28.7 (6.8)	28.9 (6.8)	0.063
○ BMI < 25.0 ○ BMI 25.0–29.9 ○ BMI ≥ 30.0	81 (38.8%)	136 (32.9%)	53 (29.9%)	0.410	80 (38.6%)	138 (33.2%)	54 (30.2%)	0.422
	69 (33.0%)	141 (34.1%)	66 (37.3%)		69 (33.3%)	140 (33.7%)	67 (37.4%)	
	59 (28.2%)	136 (32.9%)	58 (32.8%)		58 (28.0%)	138 (33.2%)	58 (32.4%)	
Total body fat content (kg)	25.3 (12.4)	27.6 (13.8)	28.2 (15.2)	0.080	25.3 (12.4)	27.5 (13.8)	28.2 (15.2)	0.095
Total body fat content (%)	30.6 (9.1)	31.8 (9.6)	31.6 (10.3)	0.377	30.6 (9.1)	31.8 (9.6)	31.6 (10.4)	0.465
Waist circumference (cm)	94.3 (17.5)	96.7 (17.2)	97.5 (16.7)	0.054	94.2 (17.6)	96.7 (17.2)	97.4 (16.6)	0.053
Hip circumference (cm)	101.3 (12.4)	104.2 (13.0)	103.8 (12.5)	*0.008*	101.2 (12.5)	104.1 (13.0)	103.8 (12.4)	*0.008*
WHR	0.927 (0.091)	0.925 (0.088)	0.937 (0.085)	0.327	0.927 (0.092)	0.925 (0.088)	0.936 (0.085)	0.382
Visceral fat (cm^3^)	103.0 (81.0)	110.0 (79.9)	112.3 (83.0)	0.379	103.3 (81.5)	110.2 (80.0)	111.8 (82.5)	0.381
Visceral fat (%)	36.4 (11.8)	37.5 (12.4)	37.2 (11.7)	0.587	36.4 (11.7)	37.6 (12.5)	37.1 (11.6)	0.570
Subcutaneous fat (cm^3^)	163.5 (83.1)	167.2 (80.5)	175.0 (82.7)	0.401	163.7 (83.5)	166.9 (80.8)	175.3 (83.1)	0.405
Subcutaneous fat (%)	63.7 (11.7)	62.3 (12.9)	62.8 (11.7)	0.557	63.7 (11.6)	62.2 (13.0)	62.9 (11.6)	0.540
Visceral/subcutaneous fat ratio	0.642 (0.406)	0.687 (0.475)	0.665 (0.413)	0.554	0.641 (0.404)	0.690 (0.477)	0.662 (0.410)	0.536
Total cholesterol (mg/dL)	202.7 (56.0)	191.7 (41.3)	194.0 (43.2)	0.070	201.9 (56.1)	192.1 (41.4)	193.7 (43.1)	0.153
HDL (mg/dL)	60.7 (14.1)	59.8 (15.6)	59.5 (14.5)	0.662	60.8 (14.0)	59.6 (15.7)	59.7 (14.4)	0.422
LDL (mg/dL)	117.3 (43.3)	109.4 (37.8)	111.2 (41.8)	0.095	116.3 (43.3)	109.9 (37.9)	111.1 (41.8)	0.189
TG (mg/dL)	123.8 (143.9)	111.9 (69.7)	116.3 (61.9)	0.289	124.1 (144.3)	113.2 (71.1)	115.1 (61.3)	0.491
Frequency of prediabetes or diabetes								
○ Yes ○ No	103 (48.8%)	209 (49.8%)	95 (52.5%)	0.751	100 (47.8%)	213 (50.2%)	96 (52.7%)	0.628
	108 (51.2%)	211 (50.2%)	86 (47.5%)		109 (52.2%)	211 (49.8%)	86 (47.3%)	
Corrected insulin response level during OGTT (10 × mU × mL × mg^−2^)								
○ 30 min ○ 60 min ○ 120 min	0.8 (0.7)	0.8 (0.7)	0.9 (1.0)	0.875	0.8 (0.7)	0.8 (0.7)	0.9 (1.0)	0.884
	0.7 (1.1)	0.6 (0.6)	0.7 (0.8)	0.325	0.7 (1.1)	0.6 (0.6)	0.7 (0.8)	0.325
	1.0 (0.7)	1.3 (1.0)	1.5 (1.6)	0.554	1.1 (0.9)	1.2 (1.0)	1.5 (1.6)	0.764
Daily energy intake (kcal)	1807.2 (732.3)	1766.9 (676.0)	1837.4 (713.4)	0.849	1820.5 (734.9)	1759.6 (673.3)	1853.9 (716.3)	0.645
Dietary fiber intake (g)	18.5 (7.2)	18.5 (8.2)	18.3 (7.6)	0.901	18.6 (7.2)	18.5 (8.2)	18.4 (7.7)	0.958
Daily physical activity level								
○ Low ○ Moderate ○ High	16 (7.6%)	25 (6.0%)	18 (9.9%)	0.302	16 (7.7%)	25 (5.9%)	19 (10.4%)	0.179
	50 (23.7%)	83 (19.8%)	40 (22.1%)		50 (23.9%)	83 (19.6%)	40 (22.0%)	
	145 (68.7%)	312 (74.3%)	123 (68.0%)		143 (68.4%)	316 (74.5%)	123 (67.6%)	

Data presented as mean and standard deviation (SD), number of observations, and frequency. BMI, body mass index; HDL, high-density lipoprotein; LDL, low-density lipoprotein; OGTT, oral glucose tolerance test. * Holm-adjusted Kruskal–Wallis/ANOVA *p* values.

**Table 2:** Characteristics of participants stratified by rs6499640 and 8044769 genotypes.

	rs6499640				rs8044769			
	A/A	A/G	G/G	*p* Value	C/C	C/T	T/T	*p* Value
N	307	377	134		270	406	138	
Genotype frequency	0.37	0.46	0.16	>0.05	0.33	0.50	0.17	>0.05
Age (years)	41.0 (14.7)	40.0 (14.2)	41.4 (15.0)	0.97	40.4 (14.8)	41.2 (14.6)	39.6 (13.7)	0.54
BMI (kg/m^2^)	28.6 (6.8)	28.6 (6.8)	27.6 (5.8)	0.358	28.5 (6.8)	28.7 (6.7)	27.8 (6.1)	0.534
○ BMI < 25.0 ○ BMI 25.0–29.9 ○ BMI ≥ 30.0	93 (30.8%)	128 (34.5%)	52 (39.4%)	0.230	88 (33.2%)	131 (32.8%)	51 (37.5%)	0.862
	118 (39.1%)	120 (32.3%)	40 (30.3%)		92 (34.7%)	143 (35.8%)	43 (31.6%)	
	91 (30.1%)	123 (33.2%)	40 (30.3%)		85 (32.1%)	126 (31.5%)	42 (30.9%)	
Total body fat content (kg)	27.5 (14.5)	27.5 (14.1)	24.9 (10.9)	0.449	27.7 (15.0)	27.2 (13.6)	25.8 (11.9)	0.676
Total body fat content (%)	31.6 (9.8)	31.6 (9.7)	30.6 (8.9)	0.597	31.7 (10.1)	31.4 (9.6)	31.1 (9.0)	0.893
Waist circumference (cm)	96.8 (17.6)	96.7 (17.4)	93.5 (15.5)	0.255	96.2 (17.2)	96.8 (17.3)	94.6 (16.8)	0.429
Hip circumference (cm)	103.4 (12.9)	104.2 (13.3)	100.7 (10.4)	0.080	103.3 (12.6)	104.0 (13.0)	101.5 (12.2)	0.189
WHR	0.933 (0.089)	0.925 (0.085)	0.925 (0.097)	0.485	0.928 (0.089)	0.927 (0.088)	0.929 (0.089)	0.980
Visceral fat (cm^3^)	109.6 (84.1)	110.2 (81.3)	101.7 (72.1)	0.822	109.2 (78.3)	110.3 (83.7)	99.8 (72.5)	0.648
Visceral fat (%)	37.3 (11.8)	36.5 (12.0)	38.2 (12.8)	0.483	37.7 (11.8)	36.9 (12.3)	36.4 (12.0)	0.617
Subcutaneous fat (cm^3^)	168.9 (85.4)	173.4 (82.6)	151.5 (70.0)	0.124	168.6 (83.3)	169.7 (82.4)	160.9 (74.2)	0.773
Subcutaneous fat (%)	62.7 (11.8)	63.3 (12.6)	61.9 (12.7)	0.503	62.3 (11.8)	63.0 (12.8)	63.7 (11.8)	0.598
Visceral/subcutaneous fat ratio	0.670 (0.410)	0.655 (0.459)	0.706 (0.472)	0.486	0.689 (0.492)	0.662 (0.421)	0.641 (0.408)	0.590
Total cholesterol (mg/dL)	195.3 (51.1)	193.8 (42.3)	196.9 (44.4)	0.646	193.8 (40.1)	191.7 (42.7)	206.7 (62.7)	*0.029*
LDL (mg/dL)	111.0 (39.6)	111.1 (40.3)	114.9 (41.7)	0.487	111.0 (38.6)	109.6 (38.7)	119.8 (46.5)	0.058
HDL (mg/dL)	60.2 (14.6)	60.3 (15.8)	58.3 (13.4)	0.289	60.7 (14.7)	59.2 (15.5)	60.4 (13.8)	0.176
TG (mg/dL)	120.5 (124.2)	111.8 (67.8)	118.6 (71.1)	0.584	110.5 (58.7)	114.6 (73.4)	132.6 (170.6)	0.689
Frequency of prediabetes or diabetes								
○ Yes ○ No	152 (49.5%)	187 (49.6%)	71 (53.0%)	0.796	143 (53.0%)	201 (49.5%)	64 (46.4%)	0.420
	155 (50.5%)	190 (50.4%)	63 (47.0%)		127 (47.0%)	205 (50.5%)	74 (53.6%)	
Corrected insulin response level during OGTT (10 × mU × mL × mg^−2^)								
○ 30 min ○ 60 min ○ 120 min	0.8 (0.9)	0.8 (0.7)	0.9 (0.8)	0.616	0.9 (0.9)	0.8 (0.7)	0.8 (0.7)	0.618
	0.6 (0.7)	0.7 (0.8)	0.7 (0.7)	0.745	0.7 (0.7)	0.7 (0.9)	0.5 (0.5)	0.194
	1.4 (1.4)	1.2 (1.0)	1.2 (1.0)	0.949	1.3 (1.3)	1.4 (1.0)	1.1 (0.8)	0.639
Daily energy intake (kcal)	1786.5 (705.2)	1825.7 (725.8)	1720.7 (594.6)	0.728	1775.7 (646.2)	1792.7 (735.8)	1816.0 (686.3)	0.791
Dietary fiber intake (g)	18.1 (7.1)	19.0 (8.5)	17.9 (7.4)	0.570	18.1 (7.4)	18.8 (8.2)	18.2 (7.4)	0.598
Daily physical activity level								
○ Low ○ Moderate ○ High	22 (7.2%)	30 (8.0%)	8 (6.0%)	0.736	23 (8.5%)	26 (6.4%)	10 (7.2%)	0.060
	68 (22.1%)	81 (21.5%)	24 (17.9%)		55 (20.4%)	77 (19.0%)	41 (29.7%)	
	217 (70.7%)	266 (70.6%)	102 (76.1%)		192 (71.1%)	303 (74.6%)	87 (63.0%)	

Data presented as mean and standard deviation (SD), number of observations, and frequency. BMI, body mass index; HDL, high-density lipoprotein; LDL, low-density lipoprotein; OGTT, oral glucose tolerance test. * Holm-adjusted Kruskal–Wallis/ANOVA *p* values.

**Table 3:** Characteristics of participants stratified by rs7190492 genotype.

	rs7190492			
	G/G	A/G	A/A	*p* Value
N	374	358	83	
Genotype frequency	0.46	0.44	0.10	>0.05
Age (years)	40.4 (14.8)	40.8 (14.4)	40.4 (13.6)	1.0
BMI (kg/m^2^)	28.7 (6.8)	28.3 (6.5)	28.1 (6.6)	0.605
○ BMI < 25.0 ○ BMI 25.0–29.9 ○ BMI ≥ 30.0	120 (32.5%)	122 (34.8%)	30 (36.1%)	0.461
	126 (34.1%)	128 (36.5%)	23 (27.7%)	
	123 (33.3%)	101 (28.8%)	30 (36.1%)	
Total body fat content (kg)	27.8 (14.5)	26.5 (13.4)	26.6 (12.4)	0.533
Total body fat content (%)	31.9 (10.0)	30.8 (9.5)	32.1 (8.8)	0.303
Waist circumference (cm)	96.6 (17.3)	96.2 (17.0)	94.7 (17.6)	0.585
Hip circumference (cm)	103.4 (12.7)	103.6 (12.9)	101.7 (12.5)	0.588
WHR	0.930 (0.090)	0.925 (0.086)	0.927 (0.092)	0.774
Visceral fat (cm^3^)	110.7 (83.0)	106.6 (81.2)	103.0 (67.1)	0.777
Visceral fat (%)	37.4 (11.6)	36.6 (12.5)	37.5 (12.5)	0.665
Subcutaneous fat (cm^3^)	170.4 (85.7)	166.5 (78.9)	160.7 (72.5)	0.921
Subcutaneous fat (%)	62.4 (12.2)	63.4 (12.5)	62.7 (12.2)	0.653
Visceral/subcutaneous fat ratio	0.674 (0.454)	0.660 (0.434)	0.676 (0.443)	0.652
Total cholesterol (mg/dL)	192.9 (40.4)	194.3 (51.0)	206.0 (47.5)	0.056
LDL (mg/dL)	110.1 (38.2)	111.4 (41.0)	119.6 (45.5)	0.219
HDL (mg/dL)	60.4 (14.6)	59.1 (15.4)	61.0 (14.0)	0.227
TG (mg/dL)	111.8 (62.8)	118.6 (119.3)	126.9 (86.2)	0.422
Frequency of prediabetes or diabetes				
○ Yes ○ No	194 (51.9%)	176 (49.2%)	38 (45.8%)	0.544
	180 (48.1%)	182 (50.8%)	45 (54.2%)	
Corrected insulin response level during OGTT (10 × mU × mL × mg^−2^)				
○ 30 min ○ 60 min ○ 120 min	0.8 (0.8)	0.9 (0.7)	0.9 (0.7)	0.421
	0.6 (0.6)	0.8 (1.0)	0.5 (0.4)	0.428
	1.3 (1.3)	1.3 (1.0)	1.2 (0.9)	0.828
Daily energy intake (kcal)	1765.4 (660.6)	1823.5 (728.6)	1771.0 (744.1)	0.609
Dietary fiber intake (g)	18.0 (6.8)	18.8 (8.7)	19.4 (8.4)	0.586
Daily physical activity level				
○ Low ○ Moderate ○ High	29 (7.8%)	23 (6.4%)	8 (9.6%)	0.626
	75 (20.1%)	75 (20.9%)	21 (25.3%)	
	270 (72.2%)	260 (72.6%)	54 (65.1%)	

Data presented as mean and standard deviation (SD), number of observations, and frequency. BMI, body mass index; HDL, high-density lipoprotein; LDL, low-density lipoprotein; OGTT, oral glucose tolerance test. * Holm-adjusted Kruskal–Wallis/ANOVA *p* values.

### 3.1. General Characteristic of Studied Population Stratified by Genotypes

Based on the food intake, physical activity, clinical, anthropometric and demographic data, we observed significant differences only in hip circumference between studied genotypes rs3751812 and rs8050136 (Table 1), and in total cholesterol level between genotypes of rs8044769 (Table 2), which was presented previously [7]. We did not observe any other significant differences between the studied genotypes.

### 3.2. Assessment of Dietary Intake

The 3-day food diaries were available exclusively from a selected subgroup of 622 participants because not all participants completed diaries properly. We did not find any differences between studied genotypes and dietary fiber intake presented in Table 1, Table 2 and Table 3. With the use of boxplots in the figures, we showed the contrast between median values of the selected responses and the interquartile ranges (IQRs) in different genotypic and dietary strata.

### 3.3. Associations between rs3751812 Polymorphism, Anthropometric Measurements, Lipid Profile, and Dietary Fiber Intake

Based on the analysis of the interactions between rs3751812 genotypes and fiber intake, we observed that GG genotype carriers presented lower hip circumferences (Figure 1A), while TT genotype carriers presented lower visceral fat content than heterozygous individuals (Figure 1B) when they were stratified to the group with higher than median fiber intake. Moreover, we noted that GG genotype carriers in the group with higher than median fiber intake presented higher total cholesterol (Figure 1C) and LDL-cholesterol levels (Figure 1D). Additionally, we observed that GG carriers showed lower corrected insulin response levels at 120 min of OGTT (Figure 1E) when they were stratified to the group with lower than median fiber intake.

### 3.4. Associations between rs8050136 Polymorphism, Anthropometric Measurements, Lipid Profile, and Dietary Fiber Intake

Analyzing the differences between rs8050136 genotypes dependent on dietary fiber intake, we observed that carriers of the CC genotype stratified to the group with higher than median fiber intake presented lower hip circumferences (Figure 2A), while AA genotype carriers presented lower visceral fat content (Figure 2B). Additionally, we observed that CC genotype carriers showed higher total cholesterol (Figure 2C) and higher LDL-cholesterol levels (Figure 2D), whereas when CC genotype carriers were stratified to the group with lower than median fiber intake, we noted lower corrected insulin response levels at 120 min of OGTT (Figure 2E).

### 3.5. Associations between rs6499640 Polymorphism, Anthropometric Measurements, Lipid Profile, and Dietary Fiber Intake

The gene–diet interaction analysis showed that GG genotype carriers stratified to the group with higher than median fiber intake presented lower hip circumferences (Figure 3A). Additionally, we observed that the AG genotype carriers presented surprisingly lower visceral fat content (Figure 3B), higher subcutaneous fat content (Figure 3C), and lower VAT/SAT ratio (Figure 3D) compared to the AA genotype carriers. We noted that homozygous AA carriers presented higher HDL-cholesterol levels (Figure 3E), and heterozygous AG carriers presented higher blood glucose levels at 60 min of OGTT (Figure 3F), compared to the heterozygous participants in the group with lower than median fiber intake. The higher levels of triglycerides were noted only in the GG genotype carriers (Figure 3G) when they were stratified to the group with higher than median fiber intake.

### 3.6. Associations between rs8044769 Polymorphism, Anthropometric Measurements, Lipid Profile, and Dietary Fiber Intake

Based on the analysis of the interactions between rs8044769 genotypes and fiber intake, we observed that TT genotype carriers, when they were stratified to higher than median fiber intake, presented lower body weight (Figure 4A) and lower fat-free mass content (Figure 4B). Moreover, we noted lower blood glucose levels at 120 min of OGTT in TT genotype carriers (Figure 4C), and lower HDL-cholesterol levels in CT genotype carriers were observed (Figure 4D) when they were stratified to the group with higher than median fiber intake.

### 3.7. Associations between rs7190492 Polymorphism, Anthropometric Measurements, Lipid Profile, and Dietary Fiber Intake

The analysis of differences between rs7190492 genotypes dependent on dietary fiber intake showed that carriers of the AG genotype stratified to the group with higher than median fiber intake presented higher fat-free mass content (Figure 5A) and higher skeletal muscle mass content (Figure 5B). We did not observe any other differences that were dependent on dietary fiber intake.

## 4. Discussion

The role of dietary habits in the prevalence and pathogenesis of obesity is significant. Comprehension of the interactions between genetic variations and dietary intake could be a solution for this dilemma. In our study, we present the association of total diet fiber intake with body composition and the subject’s glycemic and lipid profile status among Polish-origin Caucasian participants. The present study reveals that dietary fiber intake may modify the association of the *FTO* SNPs, rs3751812, rs8050136, rs9939609, rs6499640, rs8044769, and rs7190492, especially among those who consume high levels of dietary fiber. Our study demonstrated that associations of the *FTO* SNPs with hip circumference, visceral adipose tissue distribution, total cholesterol and LDL levels, and corrected insulin response levels may be dependent on daily fiber intake. In our previous study, we observed an interplay between *FTO* genetic variants and daily macronutrient intake (carbohydrates, proteins, and fat) with impact on obesity and its metabolic consequences [7]. Nevertheless, the associations between *FTO* genetic variants, dietary fiber intake, and metabolic consequences are still under investigation. Given the high prevalence of obesity in nearly all parts of Europe, and the fact that during the last four decades, the percentage of obese people substantially increased [18,19], our findings presented in this study seem to be significant for public health.

Dietary fiber is defined as a carbohydrate polymer; it is not hydrolyzed by human enzymes and is therefore not digested or absorbed from the digestive tract. Dietary fibers include soluble and insoluble fibers, and are classified based on their solubility in hot water, water holding capacity, and viscosity [20]. It is widely known that high dietary fiber intake, depending on the dietary fiber consumed, has several protective effects against chronic diseases, including diabetes, obesity, metabolic syndrome, inflammatory bowel syndrome, and cardiovascular disease [21,22,23,24]. The specific role of dietary fiber in the bioaccessibility and bioavailability of natural antioxidants derived mainly from fruits and vegetables is still widely discussed. It was shown that dietary fiber can reduce the accessibility of macronutrients, especially fat, in the human diet [25,26,27]. Moreover, dietary fiber is considered as a part of the diet that can interact with antioxidants, including polyphenol compounds. Above interaction with fiber and food components, there could be an effect of prolonged gastric emptying time and retarded absorption of nutrients in the small intestine [8].

We observed significant associations of *FTO* rs3751812 and rs8050136 according to the clinical characteristics of the studied group. Based on the anthropometric data, we noted that homozygous GG genotype carriers of rs3751812 and CC genotype carriers of rs8050136 showed significantly lower only in hip circumference. Our observations are consistent with the current state of knowledge, based on the associations of *FTO* SNPs and markers of obesity. It has been shown that the CC genotype carriers (rs8050136) present significantly lower BMI, total body fat content, and waist and hip circumferences [28,29,30]. In this case, the CC genotype carriers (rs8050136) have been shown to play a protective role, which confirms our results. Scuteri et al. [31] showed that rs3751812 was not associated with BMI and hip circumference in the population of African Americans when compared to the Hispanic and European Americans of the GenNetstudy. However, this association may be misleading due to lower minor allele frequencies or smaller effect sizes of variants in the conducted study. The fact that the effect of dietary fiber intake on the association of genotype may differ by race or ethnicity should not be ignored. For example, Villegas et al. [32] found that, in non-Hispanic whites, dietary fiber modified the association between *FTO* rs8050136 and diabetes, whereas no significant interaction was observed among non-Hispanic black participants. Taking the fact that *FTO* rs3751812 risk allele T is related to increased BMI compared to the protective allele G [33], the results that we obtained during our study seem to be appropriate. Currently, numerous studies have shown an association of *FTO* SNPs with anthropometric measurements; nevertheless, the impact of the dietary fiber intake between genotypes is still unclear.

In our study, we did not notice any crucial differences in daily dietary fiber intake between studied genotypes. However, when we stratified the study group dependent on daily fiber intake, we observed that GG genotype (rs3751812), CC genotype (rs8050136), and GG genotype (rs6499640) subjects presented lower hip circumference if daily fiber intake was higher than 18 g a day. These results are consistent with Hosseini-Esfahani et al. [34], who observed the relationship between abdominal obesity, fiber, and *FTO* rs3751812. The findings of Hosseini-Esfahani et al. suggest that individuals with a high number of risk alleles may benefit more from a higher daily dietary fiber intake versus individuals with a low number of risk alleles.

It has been noted that the *FTO* effect on obesity and other comorbidities may be modulated by a healthy dietary pattern. The traditional Mediterranean diet pattern, which may protect against type 2 diabetes [35], is low in saturated fat and includes foods rich in fiber, including vegetables, fruits, legumes, and nuts, which showed interactions with *FTO* rs9939609 [36].

Our study also showed an unexpected observation: that higher fiber intake can have a negative impact on lipid profile. However, soluble fibers have been shown to lower blood cholesterol by several mechanisms [10,37], and we observed that GG genotype (rs3751812) and CC genotype (rs8050136) subjects presented higher levels of total cholesterol and LDL-cholesterol when they were stratified to the high fiber intake quantiles. Moreover, in the same study conditions, but in the low fiber intake quantiles, we noted lower corrected insulin response levels at 120 min of OGTT in the GG genotype (rs3751812) and CC genotype (rs8050136) carriers. These observations might be associated with a source of dietary fiber and its interaction with food components, which we have not analyzed in our study. It is widely known that the source of dietary fiber has an impact on health outcomes [38]. Insoluble fibers present low or no effect on gastric emptying, macronutrient absorption, postprandial glucose responses, or blood lipid levels [39]. In contrast, consumption of soluble fibers influence serum lipids and postprandial glucose responses [40]. Freeland et al. [41] observed that higher consumption of wheat fiber intake results in increased short-chain fatty acid (SCFA) production and glucagon-like peptide-1 (GLP-1) secretion. Since GLP-1 may increase insulin sensitivity and secretion, these effects took over nine months to develop under colonic adaptation to increased wheat fiber intake.

Newly published studies showed that diets might modify the effect of the *FTO* variant on obesity, but these data are conflicting and are limited by a small sample size [42,43]. Some of the intervention studies searching for body fat distribution changes focused on diets with reduced fat or carbohydrates and increased fiber but did not find significant influence of *FTO* polymorphisms (rs8050136 and rs9939609) [44,45]. In our study, in the participants stratified to the higher daily dietary fiber intake, we found that TT genotype (rs3751812), AA genotype (rs8050136), and AG genotype (rs6499640) subjects presented lower visceral fat distribution (VAT). These results are consistent with studies focused on fiber consumption in healthy adolescent groups [46] or groups with obesity [47], but without the gene–diet analysis impact. We found no more main effects of the FTO variant on body composition during the intervention. As far as we know, there are limited studies on VAT distribution and FTO analysis, taking into consideration fiber intake. Therefore, our analysis presented in this study seems to be significant for public health awareness.

The present study has several strengths. To the best of our knowledge, this is one of the first studies to present associations between *FTO* SNPs rs3751812, rs8050136, rs9939609, rs6499640, rs7190492 and rs8044769, daily fiber intake and obesity, and glucose homeostasis and lipid profile. Moreover, our study has a relatively large sample size with excluded confounders. Another strength of our study is that we did not observe any significant differences in fiber intake between studied genotypes. This can be interpreted as a strength of our study because we can exclude the possibility of interactions between fiber and gene expression, and contribute it to consistency in gene–diet interactions. However, our study has several limitations. Our research is partially based on self-reported data, such as 3-day diaries of food intake, therefore, measurement error due to underreported or misreported data by subjects cannot be completely excluded. Nevertheless, this method is commonly being used for large-scale population investigations thus far. The other limitation is the fact that we analyzed total daily fiber intake without considering fiber source. Finally, although we obtained only Polish Caucasian individuals for our study, the data should be reflected on other populations of different ethnic groups. We included 18–79-year-old volunteers, with a mean age of around 42 years old. We did not perform analyses with an age stratification due to too small of a study group; nevertheless, it is an important factor, since most of the investigated metabolic disturbances are strongly associated with aging. Including only elderly individuals might show more significant associations, which should be considered in future analyses.

## 5. Conclusions

In summary, we found that dietary fiber intake might modify the effect of *FTO* SNPs on body composition and lipid profile. Our study indicates that daily fiber intake above 18 g per day by carriers of the GG genotype (rs3751812), CC genotype (rs8050136), and GG genotype (rs6499640) may positively affect anthropometric parameters, decreasing hip circumference, but increasing total cholesterol and LDL levels. Advances in this field bring us closer to the development of genome-customized diet recommendations to prevent obesity and its metabolic complications. Further studies are needed to verify our findings and to achieve an efficient strategy to prevent obesity development and metabolic diseases.

## Data Availability

The datasets used and/or analyzed during the current study are available from the corresponding author upon reasonable request.
